# Heart-Derived Stem Cells in Miniature Swine with Coronary Microembolization: Novel Ischemic Cardiomyopathy Model to Assess the Efficacy of Cell-Based Therapy

**DOI:** 10.1155/2016/6940195

**Published:** 2016-09-22

**Authors:** Gen Suzuki, Rebeccah F. Young, Merced M. Leiker, Takayuki Suzuki

**Affiliations:** Division of Cardiovascular Medicine, University at Buffalo Clinical and Translational Research Center, Suite 7030, 875 Ellicott Street, Buffalo, NY 14203, USA

## Abstract

A major problem in translating stem cell therapeutics is the difficulty of producing stable, long-term severe left ventricular (LV) dysfunction in a large animal model. For that purpose, extensive infarction was created in sinclair miniswine by injecting microspheres (1.5 × 10^6^ microspheres, 45 *μ*m diameter) in LAD. At 2 months after embolization, animals (*n* = 11) were randomized to receive allogeneic cardiosphere-derived cells derived from atrium (CDCs: 20 × 10^6^, *n* = 5) or saline (untreated, *n* = 6). Four weeks after therapy myocardial function, myocyte proliferation (Ki67), mitosis (phosphor-Histone H3; pHH3), apoptosis, infarct size (TTC), myocyte nuclear density, and cell size were evaluated. CDCs injected into infarcted and remodeled remote myocardium (global infusion) increased regional function and global function contrasting no change in untreated animals. CDCs reduced infarct volume and stimulated Ki67 and pHH3 positive myocytes in infarct and remote regions. As a result, myocyte number (nuclear density) increased and myocyte cell diameter decreased in both infarct and remote regions. Coronary microembolization produces stable long-term ischemic cardiomyopathy. Global infusion of CDCs stimulates myocyte regeneration and improves left ventricular ejection fraction. Thus, global infusion of CDCs could become a new therapy to reverse LV dysfunction in patients with asymptomatic heart failure.

## 1. Introduction

Cell-based regenerative therapy has emerged as a promising therapy to repair the failing heart through its potential to regenerate dead myocardium and improve left ventricular (LV) function [1–3]. Clinical trials have demonstrated the safety and feasibility of adult stem cells in humans with myocardial infarction (MI) who do not have severe heart failure [4–9]. Although intracoronary injection of cardiosphere-derived cells (CDCs) isolated from heart biopsies demonstrates promising regenerative effects in animals with myocardial infarction (MI) [3, 10, 11], clinical trials using CDCs in patients with MI did not recover global function despite an increase in viable LV tissue [8, 12]. This discrepancy of outcomes between animal studies and clinical trials is associated with the difficulty in creating optimal preclinical large animal models with severe LV dysfunction without overt heart failure symptoms since most of these clinical trials were conducted based on data from preclinical animal models with relatively preserved (EF ≈ 50%) LV dysfunction.

Since translational large animal studies of stem cell therapeutics are still limited, large animal infarction or ischemic cardiomyopathy models resembling disease conditions similar to those that occur in patients would be useful to predict the effectiveness of cell therapy strategies for clinical trials. Understanding the properties and capabilities of CDCs in MI with severe LV dysfunction or ischemic cardiomyopathy will help fill the gap between animal studies and clinical settings. We have developed a large animal model with severe LV dysfunction to treat it with cell-based therapies toward providing therapeutic insights for cardiac repair in patients with ischemic cardiomyopathy.

Microembolization in the left coronary artery of dogs, sheep, and pigs induces microinfarcts, progressive LV dilation, and stable severe LV dysfunction (EF < 40%) without overt heart failure symptom, resembling human ischemic cardiomyopathy in terms of neurohormonal activation, natriuretic peptide elaboration, myocyte hypertrophy, and interstitial fibrosis [13–17]. This model resembles ischemic cardiomyopathy or dilated ischemic cardiomyopathy in humans. While coronary microembolization in large animals recapitulates the clinical phenotype of ischemic cardiomyopathy, it did not become a popular model due to technical difficulties such as serial surgical intervention and malignant arrhythmia associated with multiple injections of microembolization [18, 19]. The majority of studies were done in canine and sheep and fewer studies were performed in swine because swine are more susceptible to ventricular arrhythmia [15, 20, 21].

Here we successfully developed a coronary microembolization procedure to create stable and severe LV dysfunction (EF < 40%) resembling human ischemic cardiomyopathy and the procedure did not cause any overt malignant arrhythmia or heart failure. Since dysfunction was stable for at least 12 weeks, we conducted studies to assess the effects of cell-based therapy between 8 and 12 weeks. We recently demonstrated that global infusion of CDCs stimulated endogenous cardiac repair systems in dysfunctional and viable myocardium or hibernating myocardium [22]. Injected CDCs regenerated myocardium in chronically ischemic as well as normally perfused remote regions. Until now, this delivery approach has never been applied to chronically infarcted myocardium. Using this model we infused CDCs in the three major coronary arteries to see the effects of regeneration in infarcted as well as viable myocardium. Accordingly, we tested whether global intracoronary infusion of CDCs regresses cellular hypertrophy and replaces myocyte loss by myocyte regeneration in infarcted and remote remodeled regions.

## 2. Materials and Methods

Experimental procedures and protocols conformed to institutional guidelines for the care and use of animals in research and were approved by the University at Buffalo Institutional Animal Care and Use Committee.

### 2.1. Microembolization Model [13, 14, 23–25]

Sinclair miniature swine (Sinclair BioResources, weight 24–36 kg, *n* = 13) were sedated with a Telazol (100 mg/mL)/xylazine (100 mg/mL) mixture (0.017 mL/lb i.m.) intubated and ventilated. Continuous sedation was maintained using an intravenous infusion of propofol (10 mg/mL at rates of 25–45 mL/hr). A 5-F Sones catheter (Cordis) was introduced via the femoral artery and the tip of the catheter was positioned in the left anterior descending coronary artery (LAD) under fluoroscopic guidance. Before microsphere injection 1.5 mg/kg of lidocaine was intravenously infused over 2-3 min. Polystyrene microspheres (Polysciences, Inc., PA, USA) were sonicated to disperse the suspension in saline with 0.05% (vol/vol) Tween 80. We injected (45 *μ*m diameter, 1.5 × 10^6^ spheres) microspheres into the proximal LAD (distal to 1st diagonal br.) to acutely abolish coronary flow reserve in the myocardium resulting in extensive myocardial necrotic lesions [26]. The microspheres were injected over 5 min under continuous monitoring of electrocardiogram and systemic blood pressure. In all animals, electrocardiographic signs of ischemia (ST-segment shifts or T-wave inversion or increased peak) were observed during the procedure, which usually resolved spontaneously within 15 min. If animals developed sustained ventricular fibrillation, they were electrically converted into sinus rhythm. Animals were group-housed in the Laboratory Animal Facility and fed a standard diet. Left ventricular dysfunction was stabilized within 60 minutes and did not lead to overt right heart failure. Physiological studies and tissue sampling were performed at selected time points.

### 2.2. Sample Collection and Processing (Cardiosphere-Derived Cell Culture) [27]

Porcine specimens were obtained from right atrium. Tissue specimens were cut into 1-2 mm^3^ pieces. After removing gross connective tissue from pieces, tissue fragments were washed and partially digested enzymatically in a solution of type IV collagenase for 60 minutes at 37°C. The tissue fragments were cultured as “explants” on dishes coated with fibronectin. After a period of 8 or more days, a layer of stromal-like cells arises from and surrounds the explants. Over this layer a population of phase-bright cells migrates. Once confluent, the cells surrounding the explants were harvested by gentle enzymatic digestion. These cardiosphere-forming cells were seeded at 1 × 10^5^ cells/mL on ultralow attachment dishes in cardiosphere medium (20% heat-inactivated fetal calf serum, 50 *μ*g/mL gentamicin, 2 mmol/L L-glutamine, and 0.1 mmol/L 2-mercaptoethanol in Iscove's modified Dulbecco medium with penicillin and streptomycin). After a period of 4–7 days in culture, cardiospheres formed and began to slowly grow in suspension. When sufficient in size and number, these free-floating cardiospheres were harvested by aspirating them along with media. Cells that remained adherent to the dishes were discarded. Detached cardiospheres were plated on fibronectin-coated flasks where they attached to the culture surface, spread out, and grew into a monolayer of cardiosphere-derived cells.

### 2.3. Serial Physiological Studies and Intracoronary CDCs Administration [28] (Figure 1)

Miniswine were brought back to the laboratory for study 2 months after microembolization. Each animal was sedated with a Telazol/xylazine mixture (0.037 mL/kg i.m.) with continuous sedation maintained using an intravenous infusion of propofol (10 mg/mL at rates of 25–45 mL/hr). Under sterile conditions, the left femoral artery was instrumented with a 6-Fr introducer. Through the introducer, a 5-Fr Millar Mikro-Tip pressure catheter was inserted into the left ventricular apex using fluoroscopic guidance. The side port of the introducer was used to monitor aortic pressure. Ear veins were used to administer propofol. The animals were heparinized (2,000 U i.v.) and hemodynamics were equilibrated for at least 30 min before the protocol was begun. After equilibration, 2D echo measurements were obtained to assess wall thickening under resting conditions. After the physiological protocol was completed, the Millar catheter was exchanged for a 5-Fr Sones catheter. Contrast left ventriculography and coronary angiography were performed to evaluate wall motion and coronary perfusion. After completing the baseline physiological measurements, allogeneic CDCs (CDCs) or saline (untreated) was administered by intracoronary infusion. Equally divided doses (total amount of 20 × 10^6^ CDCs containing 100 U/mL of heparin) were slowly injected into 3 major coronary arteries. In both CDCs and saline treated animals, cyclosporine (100 mg/day, 4 mg/kg/day) was started at 3 days prior to study and continued until the end of the study. The physiological studies were repeated 1 month after initial studies. After completing the final physiological studies, the animal was deeply anesthetized and euthanized and the heart was removed for sampling. Whole studies were conducted under investigator and technician blinded condition.

### 2.4. 2D Echocardiography

Echocardiography was used to assess regional function as previously described in pigs [29, 30]. Digitized images were obtained using a GE Vivid 7 sonography machine. The LV was imaged in the short-axis and long-axis projections from a right parasternal approach. Measurements were taken using ASE criteria. Off-axis M-mode measurements of wall thickness were obtained to calculate regional function. Systolic wall thickening (ΔWT = end-systolic wall thickness − end-diastolic wall thickness; % WT = ΔWT/end-diastolic wall thickness × 100) was measured in the dysfunctional LAD and normal remote regions. End-diastole was defined as the onset of R wave and end-systole was taken as the minimal chamber dimension during ejection. Ejection fraction was used to assess global LV function.

### 2.5. Assessment of Connective Tissue and Infarcted Area [31, 32]

The heart was weighed and sectioned into alternating 0.3 cm and 1 cm rings parallel to the AV groove from the apex to the base. Two concentric LV rings (mid-papillary muscle level and midway between the apex and the middle portion) were analyzed for histopathology. Each sample was taken from the core region of each epicardial artery perfusion territory. Histological sections were stained with a Masson-trichrome stain to contrast connective tissue staining from myocytes and connective area was quantified using pixel counting by ImageJ software. The thin concentric rings above each major ring of the left ventricle were incubated in triphenyltetrazolium chloride (TTC) to assess the extent of infarction.

### 2.6. Assessment of Myocyte Nuclear Density and Myocyte Cell Size

Briefly, tissue samples adjacent to the LAD (infarction) and the posterior descending arteries (normal) were fixed (10% formalin) and paraffin-embedded. 5 *μ*m sections were prepared for each measurement. Myocyte nuclear density and myocyte diameter were quantified using Periodic Acid-Schiff (PAS) staining [33, 34]. PAS stained sections were used to quantify myocyte diameter. Approximately 100 myocyte diameters from inner (subendocardial) and outer (epicardial) halves were averaged in LAD and normal regions.

### 2.7. Quantitation of Cell Growth/Cycle and Cell Death Markers [28, 35, 36]

To quantify myocytes in cell cycle and mitosis, paraffin-fixed tissue sections (5 *μ*m) were incubated with either anti-Ki67 (mouse monoclonal antibody, clone MIB-1, Dako) or anti-phospho-Histone H3 (rabbit polyclonal antibody, Upstate Biotech). Positive cells were visualized by Alexa Fluor 488 (Thermo Fisher). To quantify myocytes in apoptosis we used TUNEL (Chemicon Inc.) staining as previously described [34]. Myocyte nuclei were identified with cardiac Troponin I and DAPI nuclear staining. Image acquisition was performed with a multiwavelength laser confocal microscope (Zeiss LSM 700) and AxioImager equipped with ApoTome (Zeiss) as previously described [28, 37].

### 2.8. Quantitation of Capillary Density [28]

Paraffin-fixed tissue sections were incubated with Factor VIII-related antigen (Biocare Medical) followed by Alexa Fluor 488 conjugated anti-mouse antibody (Invitrogen). Nuclei were stained with DAPI. Image acquisition was performed with Zeiss's AxioImager fluorescence microscope at ×200 magnification and the number of capillaries was quantified by ImageJ software using the particle analysis feature. 10 random fields were selected and data was expressed as capillary number per tissue area (mm^2^).

## 3. Statistical Analysis

Data are expressed as mean ± standard error. A two-way ANOVA was used for the functional data, to account for the treatment effect (CDCs versus saline) and the serial studies (initial versus final). Perfusion and histological analyses were compared with a two-way ANOVA to account for treatment and region (LAD and remote region). When significant differences were detected, the Holm-Sidak test was used for all pairwise comparisons (SigmaStat 3.0). For data that was not normally distributed, square root and logarithmic transformations were performed (SigmaStat 3.0).

## 4. Results

### 4.1. Stability of LV Dysfunction in Swine with Ischemic Cardiomyopathy

**Figure 2:**
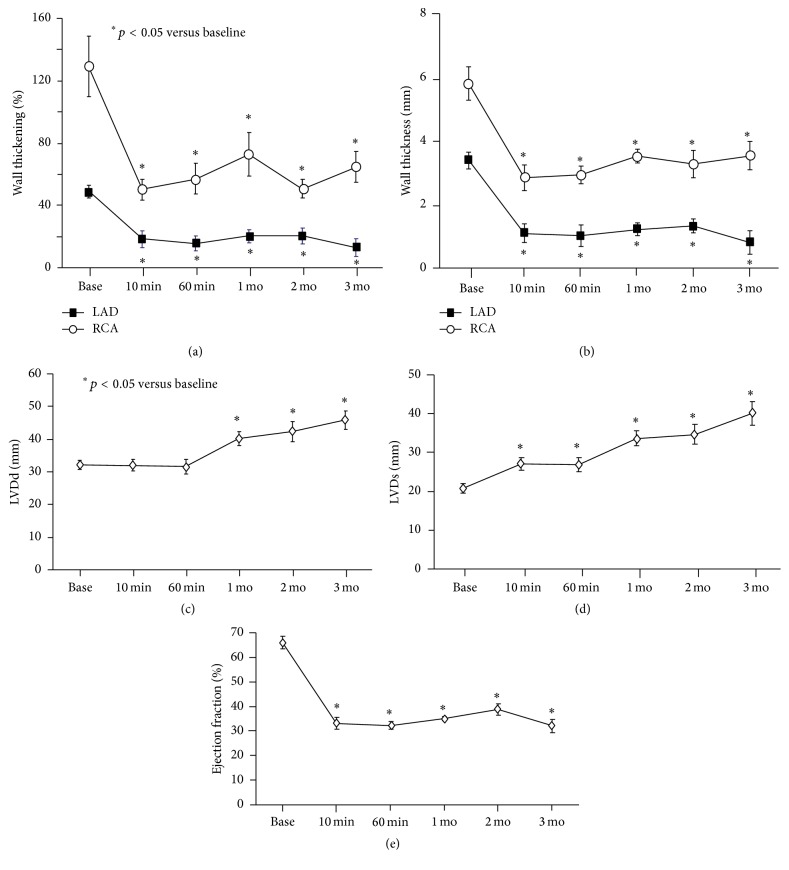
Regional and global function after microembolization. Regional and global function were severely depressed and remained constant until 3 months. The temporal changes of wall thickening (%) and Δwall thickness (mm) in the LAD and normal remote regions are summarized in (a) and (b). As early as 10 minutes after microembolization, regional wall thickening in LAD and remote regions was significantly reduced and remained constant until 3 months. Although LV end-diastolic and end-systolic dimensions slowly increased, global function remained depressed until 3 months ((c), (d), and (e)). LVDd: left ventricular distance diastole. LVDs: left ventricular distance systolic.

Thirteen pigs were embolized and two pigs died one day after embolization due to heart failure. Eleven pigs were in good health at the time of study and randomized to the study (untreated animals: *n* = 6, CDCs: *n* = 5). After coronary microembolization, physiological studies were performed at 1 month (33 ± 1 day), 2 months (66 ± 3 days), and 3 months (98 ± 4 days). None of the pigs died during the follow-up. After microembolization, regional and global function were severely depressed and remained constant over 3 months. Regional wall thickening in the LAD and normally perfused remote regions is plotted in Figures 2(a) and 2(b). As early as 10 minutes after microembolization, regional wall thickening in LAD was significantly reduced. Although wall thickening in remote regions was within normal range, wall thickening was reduced as compared to baseline and remained constant until 3-month follow-up.

Although LV end-diastolic and end-systolic dimensions were slowly increased, global function was depressed after microembolization and remained depressed until 3 months (Figures 2(c), 2(d), and 2(e)). Hemodynamic measurements of function are summarized in Table 1. LV dP/dt_Max_ was significantly decreased as early as 1 hour after coronary microembolization and remained the same until 3 months. Blood pressure was significantly decreased 1 hour after embolization but heart rate temporarily increased to maintain LV work load represented by rate pressure product. Correspondingly regional and global function were significantly reduced after microembolization and remained stable up to 3 months if intervention was not performed.

### 4.2. TTC and Connective Tissue Area

**Figure 3:**
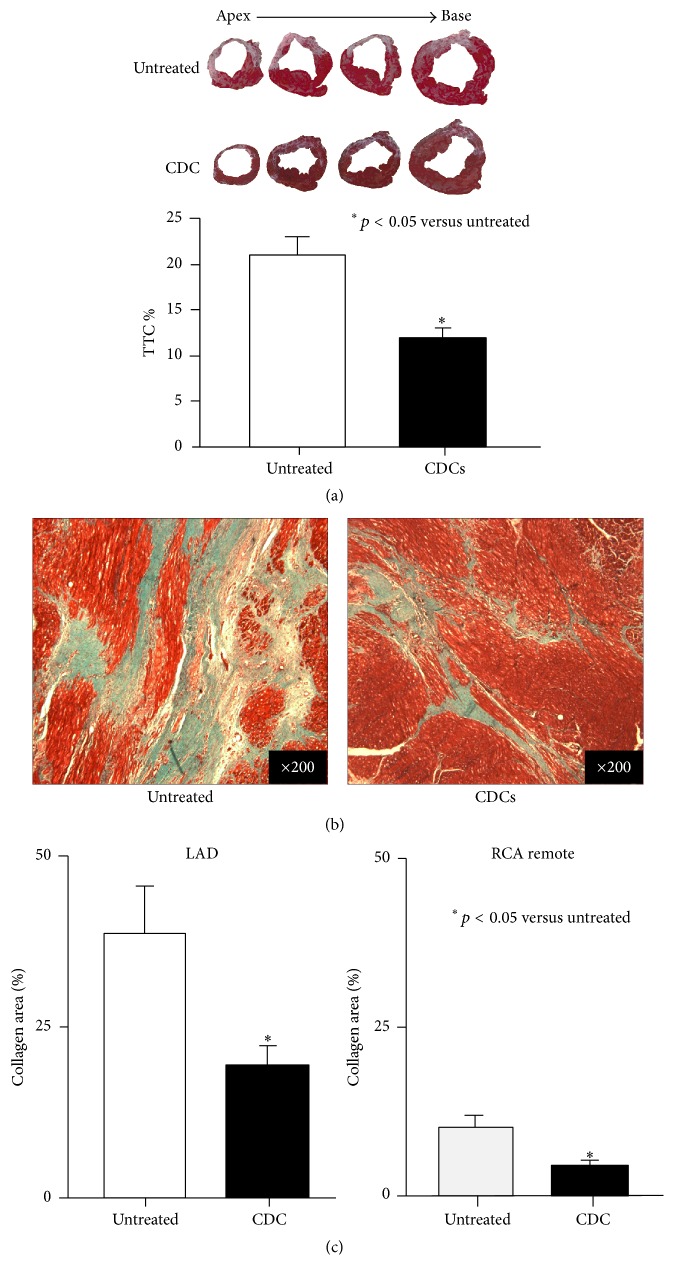
The effects of CDCs on infarction area and connective tissue area. (a) Postmortem analysis by TTC demonstrated that microembolization produced extensive myocardial infarction in anterior-septal region (21 ± 2%). Global infusion of CDCs significantly reduced infarction area (12 ± 1%). Data indicate CDC infusion reduced infarction area and increased viable myocardium. (b) Masson staining demonstrates myocardial fibrosis (blue area) in myocardium (red area). Representative images demonstrate that CDCs significantly reduced fibrotic areas compared to untreated controls. (c) Global infusion of CDCs significantly reduced LAD fibrosis (39 ± 7% to 19 ± 3%, *p* < 0.05) as well as in the RCA remote region (10 ± 2% to 5 ± 1%, *p* < 0.05).

In untreated animals, postmortem TTC staining revealed that more than 21 ± 2% of LV was infarcted. In contrast, infarction was significantly reduced in CDC animals (12 ± 1%, *p* < 0.05 versus untreated animals, Figure 3(a)). In untreated animals connective tissue area in infarcted LAD was significantly greater than remote region (39 ± 7% in infarcted LAD versus 10 ± 2 in remote region, *p* < 0.05). After CDC treatment connective tissue was significantly reduced in both infarcted LAD and remote regions (19 ± 3% and 5 ± 1%, *p* < 0.05 versus untreated animals, resp., Figure 3(b)). Data indicate that CDC infusion reduced scar volume and connective tissue area in both infarcted LAD and remote regions.

### 4.3. Effects of CDCs on Function and Flow in Ischemic Cardiomyopathy

**Figure 4:**
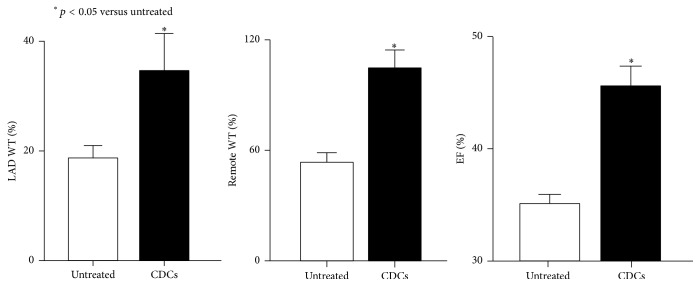
The effect of CDCs on cardiac function. In untreated controls, regional and global function assessed by echocardiography were severely reduced. After 4 weeks, CDCs significantly improved LAD and remote wall thickening, producing an improvement in LV ejection fraction.

Hemodynamic and echocardiographic measurements of function are summarized in Tables 2 and 3. LV dP/dt_Max_ was significantly increased after CDCs (*p* < 0.05 versus untreated animals), as was LV dP/dt_Min_. CDCs increased systolic wall thickening in infarcted LAD and remote regions (percent WT: 16 ± 6% to 36 ± 6% in infarcted LAD and 51 ± 5% to 99 ± 10% in remote regions, *p* < 0.05 versus initial and untreated animals) summarized in Figure 4 and Table 3. Global function was significantly increased after CDCs (ejection fraction: 29 ± 3% to 45 ± 4%, *p* < 0.05 versus initial and untreated animals) while EF remained constant in untreated animals (39 ± 2% to 32 ± 3%, *p*-ns).

### 4.4. Effects of CDCs on Proliferative Myocytes, Myocyte Mitosis, and Apoptosis in Ischemic Cardiomyopathy

**Figure 5:**
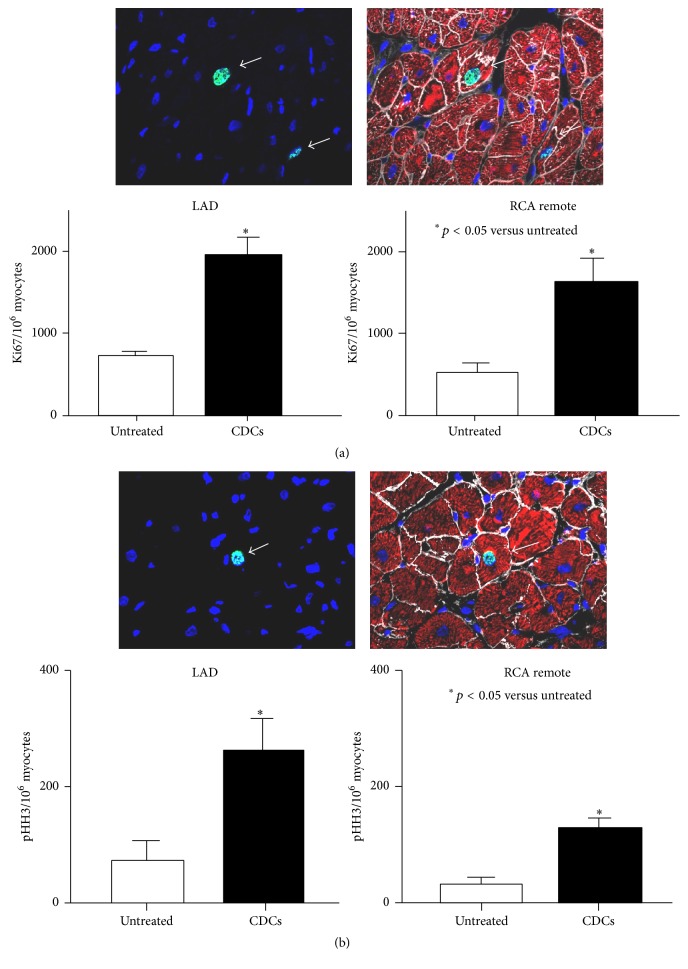
The effect of CDCs on proliferating myocytes. (a) The upper panel shows a Ki67 positive nucleus localized in a cardiac myocyte. Two Ki67 positive nuclei (green, arrows) colocalized with myocytes stained with Troponin I (red). The number of cardiac myocytes in the growth phase of the cell cycle was evaluated with Ki67. Four weeks after CDCs, Ki67 positive myocytes were significantly increased in both LAD and RCA remote regions versus untreated controls. (b) pHH3 positive nuclei (green, arrow) colocalized with myocytes (red). Cardiac myocytes in the mitotic phase were evaluated with phospho-Histone H3 (pHH3). Four weeks after CDCs, myocyte nuclei in the mitotic phase significantly increased in both LAD and RCA remote regions versus untreated controls.

We quantified the frequency of myocyte nuclei expressing Ki67, a marker of the cell cycle, and phospho-Histone H3, a marker of mitosis (Figures 5(a) and 5(b)). After CDCs, Ki67 positivity increased (infarcted LAD: 1952 ± 215 in CDCs versus 745 ± 77 nuclei per 10^6^ myocyte nuclei in untreated animals, *p* < 0.05). Likewise, myocyte nuclear phospho-Histone H3 (pHH3) positivity was significantly increased in CDCs animals (infarcted LAD: 263 ± 55 in CDCs versus 61 ± 25 nuclei per 10^6^ myocytes nuclei in untreated animals, *p* < 0.05). In remote regions Ki67 positive myocytes increased after CDCs (remote regions: 1577 ± 295 in CDCs versus 477 ± 94 nuclei per 10^6^ myocyte nuclei in untreated animals, *p* < 0.05). pHH3 positive myocytes increased in CDC animals (remote regions: 130 ± 17 in CDCs versus 32 ± 11 nuclei per 10^6^ myocyte nuclei in untreated animals, *p* < 0.05).

**Figure 6:**
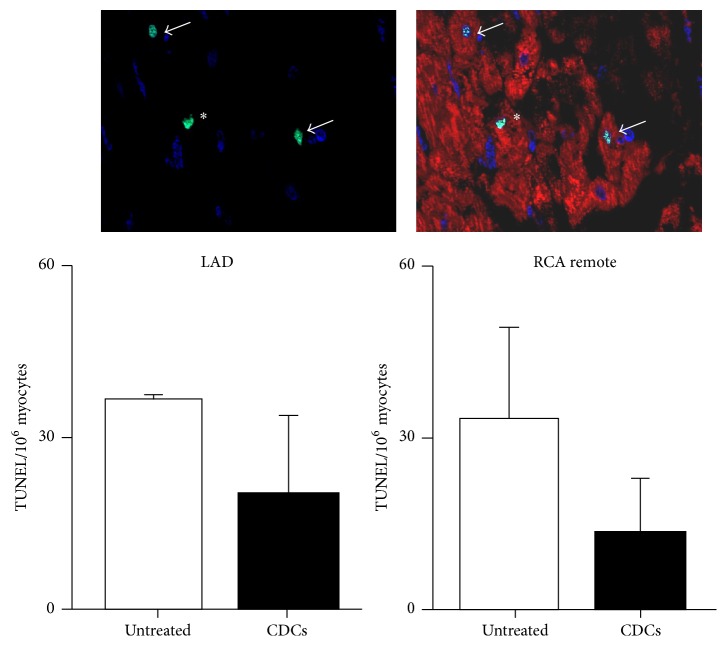
The effects of CDCs on TUNEL positive myocytes. The upper panel represents TUNEL positive nuclei localized in cardiac myocytes. Two TUNEL positive nuclei (green, arrow) localized to myocytes by cTnI staining (red). One TUNEL positive nonmyocyte nucleus located in the interstitial space (*∗*). The lower graph summarizes the number of TUNEL positive myocytes detected in the heart, expressed as nuclei per million myocytes. After CDCs, there was no significant change in the number of TUNEL positive myocytes in ischemic LAD and remote regions.

We also quantified the effects of CDCs on myocyte apoptosis in ischemic cardiomyopathy (Figure 6). There was a trend toward the reduction of apoptotic TUNEL positive myocytes in ischemic cardiomyopathy after CDC treatment as compared to untreated animals in both ischemic and remote regions, but it did not reach significance (20 ± 13 in CDCs versus 49 ± 17 nuclei per 10^6^ myocytes in untreated animals, *p* = 0.08, 14 ± 9 in CDCs versus 41 ± 10 nuclei per 10^6^ myocytes in untreated animals, *p* = 0.09).

### 4.5. Effects of CDCs on Myocyte Nuclear Density and Myocyte Diameter in Ischemic Cardiomyopathy

**Figure 7:**
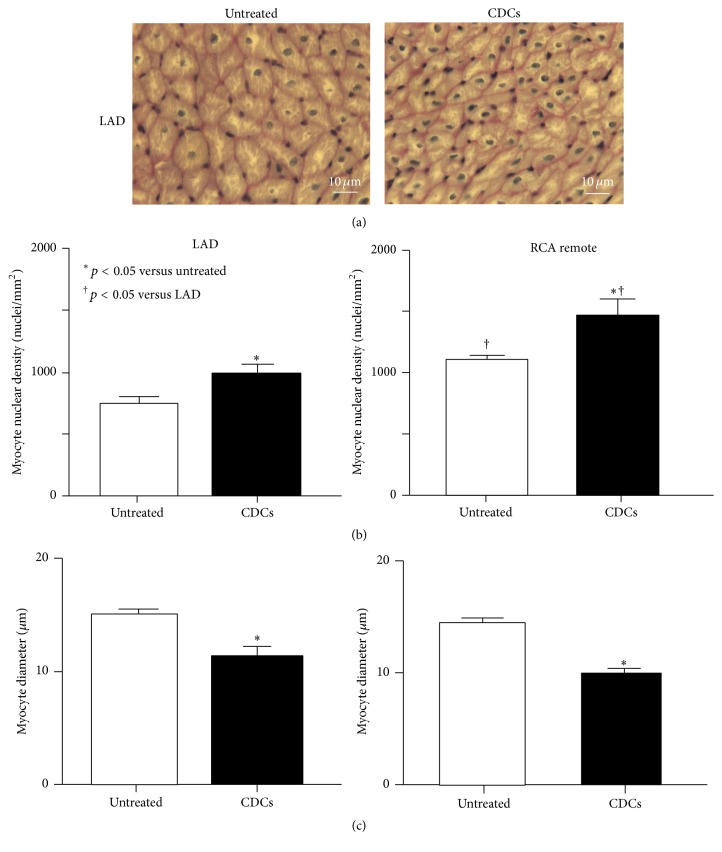
The effects of CDCs on myocyte number and myocyte size. (a) Upper images represent PAS stained cardiac myocytes in LAD region from untreated and CDC treated animals. (b) Myocyte nuclear density was quantified as a cumulative index of myocyte regeneration following CDC infusion. In untreated animals, myocyte nuclear density was significantly reduced, reflecting infarct-related myocyte loss. After CDCs there was a significant increase in myocyte nuclear density in LAD and RCA remote regions. (c) The myocyte regeneration that resulted from CDCs led to a global reduction in myocyte diameter as compared to untreated controls. There was a significant reduction in myocyte size in both LAD and RCA remote regions. This supports the formation of new myocytes as a cause of the global functional improvement.

To determine whether increased proliferative myocytes lead to myocyte regeneration, we assessed the effects of CDCs on myocyte nuclear density and myocyte cell size (Figure 7). In untreated animals, LAD myocyte nuclear density was significantly reduced compared to normal remote regions (infarcted LAD 686 ± 54 versus remote regions 1066 ± 84 nuclei/mm^2^, *p* < 0.05). Myocyte nuclear density in infarcted LAD regions was increased with CDCs (992 ± 55 nuclei/mm^2^, *p* < 0.01 versus untreated animals). In remote regions myocyte nuclear density was significantly increased as compared to untreated animals (1478 ± 98 nuclei/mm^2^ in CDCs versus untreated animals, *p* < 0.01).

In untreated animals, myocyte diameter in the infarcted LAD region was significantly greater than that in the normal remote region (14.8 ± 0.7 *μ*m in LAD versus 13.5 ± 0.5 *μ*m in remote region, *p* < 0.05) indicating myocyte hypertrophy due to myocyte loss in ischemic cardiomyopathy (Figure 7). Myocyte diameter was significantly reduced in CDCs animals (11.8 ± 0.2 *μ*m in CDCs versus untreated animals, *p* < 0.05) accompanied by an increase in nuclear density. Likewise, myocyte diameter in the remote region was significantly reduced in CDCs as compared to untreated animals (10.4 ± 0.1 *μ*m in CDCs, *p* < 0.05). Taken together, this suggests an increase in myocyte nuclear density and a reduction in myocyte size resulting from myocyte proliferation after CDCs treatment for ischemic cardiomyopathy.

### 4.6. Effects of CDCs on Capillary Density in Ischemic Cardiomyopathy

**Figure 8:**
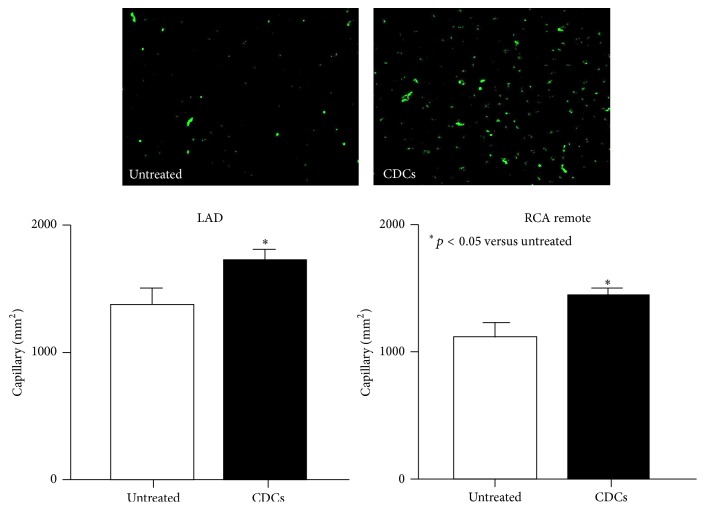
The effect of CDCs on capillary density. The upper panel represents images of capillary vessels stained with von Willebrand in ischemic myocardium. vWF positive cells were localized with capillary vessels as shown in green. DAPI nuclei are in blue. The lower graphs summarize the number of vWF positive capillaries (number per mm^2^). CDCs increased capillary density indicating angiogenesis. These data indicate that CDCs increased angiogenesis as well as myocyte regeneration in swine with ischemic cardiomyopathy.

Corresponding to myocyte regeneration, CDCs stimulated angiogenesis by increasing capillary density in infarcted LAD (Figure 8, 1735 ± 75 in CDCs to 1236 ± 144/mm^2^ in untreated animals, *p* < 0.05). In the remote region, CDCs increased capillary density (1382 ± 54 in CDCs to 1023 ± 73/mm^2^ in untreated animals, *p* < 0.05). These data indicate that CDCs increased angiogenesis accompanied by myocyte production in swine with ischemic cardiomyopathy.

## 5. Discussion

We demonstrated that a single injection of large diameter microspheres in miniswine created long-term stable and extensive LV dysfunction without signs of decompensated heart failure. The procedure is simple and perioperative mortality is minimal. Therefore, it can be used to create a preclinical large animal model of ischemic cardiomyopathy. Using this model we evaluated the beneficial effects of global intracoronary infusion of CDCs. (1) It amplifies the number of Ki67 and pHH3 positive myocytes in ischemic and remote regions. (2) It reduces myocyte size and increases myocyte nuclear density, suggesting myocyte regeneration in ischemic and remote regions. (3) CDCs stimulated angiogenesis in the ischemic area as well as normally perfused remote regions. Although longer-term follow-up is required, the data supports the hypothesis that CDCs activate the endogenous cardiac repair system in the entire heart during ischemic cardiomyopathy.

### 5.1. The Importance of Preclinical Model with Stable and Severe LV Dysfunction

A major problem in translating stem cell therapeutics is the inability to produce stable, long-term ischemic cardiomyopathy in a large animal model. Postinfarction rodent models of heart failure are easily developed since rats and mice regularly survive infarcts exceeding 50% of LV mass. These have not been uniformly duplicated in large animals since, similar to humans, large animals have a high mortality when the infarct size exceeds 30% of LV mass while smaller infarcts (<20% of LV mass) have a normal ejection fraction without heart failure. Therefore, controlling infarction size between 20 and 30% of LV mass is a major challenge in current studies.

Our coronary microembolization model created severe LV dysfunction (EF: 31 ± 2%) in as early as 10 minutes, with dysfunction maintained up to 3 months (EF: 32 ± 3%). Although this approach resulted in an infarction that averages approximately 20% of LV mass, mortality rate was limited to 15% (2 out of 13 pigs). We initiated cell therapy at 2 months after microembolization and performed follow-up one month later. Since miniswine grow slower than Yorkshire farm-bred pigs, we will be able to conduct longer follow-up (>6 months) in the future.

This model can also be used in conjunction with other disease conditions to more closely mimic real human situations. Most of large animal studies use otherwise healthy pigs which do not have common cardiovascular risk factors such as hypercholesterolemia and diabetes. We anticipate that minipigs closely resembling human disease conditions will be useful to predict the effectiveness of cell therapy in subsequent clinical trials.

### 5.2. CDCs Stimulate Cardiomyocytes in Proliferation and Increased Newly Formed Myocytes

CDCs enhanced the number of Ki67 and pHH3 positive cardiomyocytes. These changes are associated with increased nuclear density and the formation of cardiomyocytes with small diameters. Recently, genetic fate-mapping experiments demonstrated that myocyte regeneration is very low in normal adult hearts but myocardial infarction stimulates myocyte proliferation and the differentiation of endogenous progenitor cells to promote cardiac repair [36, 38, 39]. Furthermore, cell therapy using cardiac stem cells (cardiosphere-derived cells) amplifies both myocyte proliferation and the differentiation of endogenous cardiac progenitor cells [40]. Our data indicate that increases in Ki67 positive cardiomyocytes were in good correlation in both ischemic and nonischemic regions, implicating cardiomyocyte proliferation and endogenous cardiac stem cell differentiation for cardiac regeneration. Previously we demonstrated that the increase in pHH3 positive myocytes correlated with an increase in cardiomyocytes with single nuclei after intracoronary injection of CDCs in hibernating myocardium [18]. CDCs increased pHH3 positive myocytes and the net number of myocytes, but the number of binucleated myocytes was not altered. This data indicates that myocyte mitosis preferentially creates myocytes with a single nucleus. Future studies will track cell fate and confirm the contribution of endogenous cardiac stem cells and proliferating cardiomyocytes, using genetic fate-mapping [34] or bone marrow transplantation [36].

### 5.3. The Effect of CDCs on Reversing Cardiac Hypertrophy

Besides their regeneration potential, CDCs may have potential to induce hypertrophy regression in preexisting myocytes. We previously demonstrated that global infusion of CDCs into hibernating myocardium significantly increased small myocytes in the ischemic and nonischemic regions [22]. Interestingly, CDCs also reduced hypertrophic signaling (mitogen activated kinases) in the ischemic and nonischemic regions [41]. Data indicate that CDCs have the potential to reverse cardiac hypertrophy. Whether hypertrophy regression is primary or secondary to myocardial regeneration by creating newly formed small myocytes will be addressed in future studies.

### 5.4. The Effects of CDCs on Myocyte Apoptosis and Angiogenesis

Myocyte apoptosis was low in untreated animals 3 months after microembolization. CDCs tended to reduce myocyte apoptosis further, but there was no significant difference. Although we did not measure temporal changes in myocyte apoptosis after microembolization, we assume the majority of myocyte death associated with acute ischemia was completed at the acute phase of microembolization with little progressive change for 2 and 3 months. Since hemodynamics are stabilized at 2 months, other apoptotic pathways related to stretch or inflammation may not be activated.

CDCs increased capillary density as compared to untreated animals suggesting their role in neovascularization to improve cardiac function in cardiomyopathy. Increases in angiogenesis were accompanied by increases in myocyte regeneration. These changes correspond to a decrease in myocardial apoptosis and contribute to the recovery of cardiac function.

### 5.5. Cardioprotective Effect of Cyclosporine

Although it is reported that allogeneic CDCs can escape from the immune-rejection, we used cyclosporine or T cell suppressors to alleviate nonspecific immune-response associated with allogeneic stem cell injection in this study. Since cyclosporine administration is also known to have pleiotropic protective effect on myocardial damage after acute myocardial infarction [42–44], cyclosporine may reduce infarct size and preserve cardiac function. However, in this study we administered cyclosporine 2 months after microembolization with both CDC treated and untreated animals receiving equal treatment. Thus, we believe cyclosporine did not affect the outcomes in the current study.

In summary, our data confirms the ability of endogenous progenitor cells to participate in myocyte regeneration to repair the damaged heart when triggered by stem cell injections. This successful therapeutic approach will be proposed as an alternative therapy for patients with ischemic and nonischemic cardiomyopathy who are inoperable or have dysfunction due to massive infarction.

## Figures and Tables

**Figure 1:**
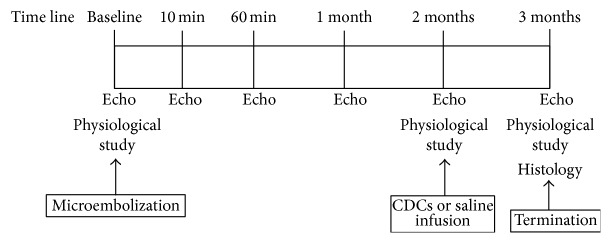
Study protocol for microembolization and CDC infusion.

**Table 1:** The effects of coronary microembolization on hemodynamics.

	Systolic pressure (mmHg)	Mean aortic pressure (mmHg)	Heart rate (bpm)	LVEDP (mmHg)	LV dP/dt_Max_ (mmHg/sec)	RPP
Baseline	135 ± 8	107 ± 6	86 ± 7	20 ± 3	2348 ± 64	11454 ± 731
1 hour	107 ± 12^*∗*^	83 ± 9^*∗*^	100 ± 7	18 ± 3	1336 ± 81^*∗*^	10728 ± 1317
2 months	144 ± 7	116 ± 6	77 ± 5	28 ± 3	1537 ± 33^*∗*^	10944 ± 599
3 months	140 ± 8	113 ± 7	88 ± 8	26 ± 2	1437 ± 184^*∗*^	12832 ± 1683

Values are mean ± SEM; ^*∗*^ *p* < 0.05 versus baseline, LVEDP: left ventricular end-diastolic pressure, LV dP/dt: first derivative of LV pressure, and RPP: rate pressure product.

**Table 2:** The effects of CDCs on hemodynamics.

	Systolic pressure (mmHg)	Mean aortic pressure (mmHg)	Heart rate (bpm)	LVEDP (mmHg)	LV dP/dt_Max_ (mmHg/sec)	LV dP/dt_Min_ (mmHg/sec)
*Untreated animals*						
Initial	144 ± 10	116 ± 7	77 ± 6	28 ± 3	1537 ± 40	−1964 ± 78
Final	140 ± 10	113 ± 8	88 ± 10	26 ± 3	1222 ± 126	−1510 ± 120
*CDCs*						
Initial	127 ± 6	93 ± 5	69 ± 6	28 ± 2	1329 ± 103	−1969 ± 261
Final	133 ± 3	112 ± 4	85 ± 6	25 ± 2	1613 ± 90^*∗*†^	−1525 ± 735

Values are mean ± SEM; LVEDP: left ventricular end-diastolic pressure; ^*∗*^ *p* < 0.05 versus initial; ^†^ *p* < 0.05 versus untreated animals; untreated animals *n* = 6; and CDCs *n* = 5.

**Table 3:** The effects of CDCs on echocardiographic measurements.

	*n*	LAD WT (%)	Remote WT (%)	FS (%)	EF (%)
*Untreated animals*					
Initial	6	20 ± 4	58 ± 4	18 ± 1	39 ± 2
Final		13 ± 6	47 ± 8	13 ± 2^*∗*^	32 ± 3^*∗*^
*CDCs*					
Initial	5	16 ± 6	51 ± 5	13 ± 1	29 ± 3
Final		36 ± 6^*∗*†^	99 ± 10^*∗*†^	21 ± 2^*∗*†^	45 ± 4^*∗*†^

Values are mean ± SEM; ^*∗*^ *p* < 0.05 versus initial; ^†^ *p* < 0.05 versus untreated animals; LAD: left anterior descending coronary artery; WT: wall thickening; FS: fractional shortening; and EF: ejection fraction.

## References

[1] Schuleri K. H., Feigenbaum G. S., Centola M. (2009). Autologous mesenchymal stem cells produce reverse remodelling in chronic ischaemic cardiomyopathy. *European Heart Journal*.

[2] Hatzistergos K. E., Quevedo H., Oskouei B. N. (2010). Bone marrow mesenchymal stem cells stimulate cardiac stem cell proliferation and differentiation. *Circulation Research*.

[3] Johnston P. V., Sasano T., Mills K. (2009). Engraftment, differentiation, and functional benefits of autologous cardiosphere-derived cells in porcine ischemic cardiomyopathy. *Circulation*.

[4] Schächinger V., Assmus B., Britten M. B. (2004). Transplantation of progenitor cells and regeneration enhancement in acute myocardial infarction: final one-year results of the TOPCARE-AMI trial. *Journal of the American College of Cardiology*.

[5] Hare J. M., Fishman J. E., Gerstenblith G. (2012). Comparison of allogeneic vs autologous bone marrow-derived mesenchymal stem cells delivered by transendocardial injection in patients with ischemic cardiomyopathy: the POSEIDON randomized trial. *The Journal of the American Medical Association*.

[6] Williams A. R., Trachtenberg B., Velazquez D. L. (2011). Intramyocardial stem cell injection in patients with ischemic cardiomyopathy: functional recovery and reverse remodeling. *Circulation Research*.

[7] Chugh A. R., Beache G. M., Loughran J. H. (2012). Administration of cardiac stem cells in patients with ischemic cardiomyopathy: the SCIPIO trial: surgical aspects and interim analysis of myocardial function and viability by magnetic resonance. *Circulation*.

[8] Makkar R. R., Smith R. R., Cheng K. (2012). Intracoronary cardiosphere-derived cells for heart regeneration after myocardial infarction (CADUCEUS): a prospective, randomised phase 1 trial. *The Lancet*.

[9] Bolli R., Chugh A. R., D'Amario D. (2011). Cardiac stem cells in patients with ischaemic cardiomyopathy (SCIPIO): initial results of a randomised phase 1 trial. *The Lancet*.

[10] Chimenti I., Smith R. R., Li T.-S. (2010). Relative roles of direct regeneration versus paracrine effects of human cardiosphere-derived cells transplanted into infarcted mice. *Circulation Research*.

[11] Li T.-S., Cheng K., Malliaras K. (2012). Direct comparison of different stem cell types and subpopulations reveals superior paracrine potency and myocardial repair efficacy with cardiosphere-derived cells. *Journal of the American College of Cardiology*.

[12] Malliaras K., Makkar R. R., Smith R. R. (2014). Intracoronary cardiosphere-derived cells after myocardial infarction: evidence of therapeutic regeneration in the final 1-year results of the CADUCEUS trial (cardiosphere-derived autologous stem cells to reverse ventricular dysfunction). *Journal of the American College of Cardiology*.

[13] Heusch G., Schulz R., Haude M., Erbel R. (2004). Coronary microembolization. *Journal of Molecular and Cellular Cardiology*.

[14] Skyschally A., Leineweber K., Gres P., Haude M., Erbel R., Heusch G. (2006). Coronary microembolization. *Basic Research in Cardiology*.

[15] Yarbrough W. M., Spinale F. G. (2003). Large animal models of congestive heart failure: a critical step in translating basic observations into clinical applications. *Journal of Nuclear Cardiology*.

[16] Thielmann M., Dörge H., Martin C. (2002). Myocardial dysfunction with coronary microembolization: signal transduction through a sequence of nitric oxide, tumor necrosis factor-*α*, and sphingosine. *Circulation Research*.

[17] Canton M., Skyschally A., Menabò R. (2006). Oxidative modification of tropomyosin and myocardial dysfunction following coronary microembolization. *European Heart Journal*.

[18] Heusch G., Kleinbongard P., Böse D. (2009). Coronary microembolization: from bedside to bench and back to bedside. *Circulation*.

[19] Sabbah H. N., Chandler M. P., Mishima T. (2002). Ranolazine, a partial fatty acid oxidation (pFOX) inhibitor, improves left ventricular function in dogs with chronic heart failure. *Journal of Cardiac Failure*.

[20] Carlsson M., Martin A. J., Ursell P. C., Saloner D., Saeed M. (2009). Magnetic resonance imaging quantification of left ventricular dysfunction following coronary microembolization. *Magnetic Resonance in Medicine*.

[21] Houser S. R., Margulies K. B., Murphy A. M. (2012). Animal models of heart failure: a scientific statement from the American Heart Association. *Circulation Research*.

[22] Suzuki G., Weil B. R., Leiker M. M. (2014). Global intracoronary infusion of allogeneic cardiosphere-derived cells improves ventricular function and stimulates endogenous myocyte regeneration throughout the heart in swine with hibernating myocardium. *PLoS ONE*.

[23] Heusch G., Schulz R. (2001). Perfusion-contraction match and mismatch. *Basic Research in Cardiology*.

[24] Sabbah H. N., Sharov V. G., Gupta R. C., Todor A., Singh V., Goldstein S. (2000). Chronic therapy with metoprolol attenuates cardiomyocyte apoptosis in dogs with heart failure. *Journal of the American College of Cardiology*.

[25] Schmermund A., Lerman L. O., Rumberger J. A. (2000). Effects of acute and chronic angiotensin receptor blockade on myocardial vascular blood volume and perfusion in a pig model of coronary microembolization. *American Journal of Hypertension*.

[26] Skyschally A., Schulz R., Erbel R., Heusch G. (2002). Reduced coronary and inotropic reserves with coronary microembolization. *American Journal of Physiology—Heart and Circulatory Physiology*.

[27] Smith R. R., Barile L., Cho H. C. (2007). Regenerative potential of cardiosphere-derived cells expanded from percutaneous endomyocardial biopsy specimens. *Circulation*.

[28] Suzuki G., Iyer V., Lee T.-C., Canty J. M. (2011). Autologous mesenchymal stem cells mobilize cKit^+^ and CD133^+^ bone marrow progenitor cells and improve regional function in hibernating myocardium. *Circulation Research*.

[29] Malm B. J., Suzuki G., Canty J. M., Fallavollita J. A. (2002). Variability of contractile reserve in hibernating myocardium: dependence on the method of inotropic stimulation. *Cardiovascular Research*.

[30] Fallavollita J. A., Canty J. M. (2002). Ischemic cardiomyopathy in pigs with two-vessel occlusion and viable, chronically dysfunctional myocardium. *American Journal of Physiology—Heart and Circulatory Physiology*.

[31] Fallavollita J. A., Perry B. J., Canty J. M. (1997). ^18^F-2-deoxyglucose deposition and regional flow in pigs with chronically dysfunctional myocardium: evidence for transmural variations in chronic hibernating myocardium. *Circulation*.

[32] Fallavollita J. A., Logue M., Canty J. M. (2001). Stability of hibernating myocardium in pigs with a chronic left anterior descending coronary artery stenosis: absence of progressive fibrosis in the setting of stable reductions in flow, function and coronary flow reserve. *Journal of the American College of Cardiology*.

[33] Suzuki G., Lee T.-C., Fallavollita J. A., Canty J. M. (2005). Adenoviral gene transfer of FGF-5 to hibernating myocardium improves function and stimulates myocytes to hypertrophy and reenter the cell cycle. *Circulation Research*.

[34] Hajin L., Fallavollita J. A., Hard R., Kerr C. W., Canty J. M. (1999). Profound apoptosis-mediated regional myocyte loss and compensatory hypertrophy in pigs with hibernating myocardium. *Circulation*.

[35] Lynch P., Lee T.-C., Fallavollita J. A., Canty J. M., Suzuki G. (2007). Intracoronary administration of AdvFGF-5 (fibroblast growth factor-5) ameliorates left ventricular dysfunction and prevents myocyte loss in swine with developing collaterals and ischemic cardiomyopathy. *Circulation*.

[36] Hsieh P. C. H., Segers V. F. M., Davis M. E. (2007). Evidence from a genetic fate-mapping study that stem cells refresh adult mammalian cardiomyocytes after injury. *Nature Medicine*.

[37] Suzuki G., Iyer V., Cimato T., Canty J. M. (2009). Pravastatin improves function in hibernating myocardium by mobilizing CD133^+^ and cKit^+^ bone marrow progenitor cells and promoting myocytes to reenter the growth phase of the cardiac cell cycle. *Circulation Research*.

[38] Loffredo F. S., Steinhauser M. L., Gannon J., Lee R. T. (2011). Bone marrow-derived cell therapy stimulates endogenous cardiomyocyte progenitors and promotes cardiac repair. *Cell Stem Cell*.

[39] Senyo S. E., Steinhauser M. L., Pizzimenti C. L. (2013). Mammalian heart renewal by pre-existing cardiomyocytes. *Nature*.

[40] Tseliou E., Pollan S., Malliaras K. (2013). Allogeneic cardiospheres safely boost cardiac function and attenuate adverse remodeling after myocardial infarction in immunologically mismatched rat strains. *Journal of the American College of Cardiology*.

[41] Suzuki G. (2015). Translational research of adult stem cell therapy. *World Journal of Cardiology*.

[42] Piot C., Croisille P., Staat P. (2008). Effect of cyclosporine on reperfusion injury in acute myocardial infarction. *The New England Journal of Medicine*.

[43] Jansen of Lorkeers S. J., Hart E., Tang X. L. (2014). Cyclosporin in cell therapy for cardiac regeneration. *Journal of Cardiovascular Translational Research*.

[44] Heusch G. (2015). CIRCUS: a kiss of death for cardioprotection?. *Cardiovascular Research*.

